# Pericranial Muscle Stiffness, Pain Thresholds, and Tenderness during a Treatment Cycle of OnabotulinumtoxinA for Chronic Migraine Prevention

**DOI:** 10.3390/diagnostics14030330

**Published:** 2024-02-03

**Authors:** Sebastian Worsaae Dalby, Jeppe Hvedstrup, Louise Ninett Carlsen, Sait Ashina, Lars Bendtsen, Henrik Winther Schytz

**Affiliations:** 1 Danish Headache Center, Department of Neurology, Copenhagen University Hospital–Rigshospitalet-Glostrup, 2600 Copenhagen, Denmark; sebastian.worsaae.dalby.01@regionh.dk (S.W.D.); jeppe.hvedstrup.mann@regionh.dk (J.H.);; 2 Department of Clinical Medicine, Faculty of Health and Medical Sciences, University of Copenhagen, 1871 Copenhagen, Denmark; 3 Comprehensive Headache Center, Department of Neurology, Department of Anesthesia, Critical Care and Pain Medicine, Beth Israel Deaconess Medical Center, Harvard Medical School, Boston, MA 02115, USA

**Keywords:** chronic migraine, OnabotulinumtoxinA, headache

## Abstract

Background: Treatment with OnabotulinumtoxinA (BoNT-A) is effective as a preventive treatment for chronic migraine (CM). Preclinical studies suggest that the mechanism of action of BoNT-A in migraine is based on blocking unmyelinated C fibers. We aimed to investigate whether the muscle-relaxing effect of BoNT-A is associated with the preventive mechanism in patients with chronic migraine by measuring the stiffness, pain thresholds, and tenderness of the BoNT-A-applied muscles. Methods: A total of 22 patients with CM who were already in BoNT-A treatment participated in this longitudinal prospective study. Pericranial muscle stiffness was measured using ultrasound shear wave elastography, which measures the speed of shear waves propagating through the muscle. Pressure pain thresholds (PPT) were obtained via algometry, and muscle tenderness was measured via manual palpation. Measurements were made before BoNT-A injections and six weeks after the treatment. The measurements were performed while the muscles were maximally relaxed. The patients also completed daily diaries on headache and neck pain. Results: No change was observed in muscle stiffness (*p* = 0.737) or pericranial muscle tenderness (*p* = 0.400). The PPT over the trapezius muscles increased from 250 kPa before treatment to 304 kPa six weeks after treatment (*p* = 0.027). No change was observed on the temporalis muscles (*p* = 0.200) nor the non-dominant index finger (*p* = 0.067). BoNT-A decreased neck pain (*p* = 0.008) and headache (*p* = 0.007). Conclusions: The findings suggest that BoNT-A leads to the desensitization of cutaneous and muscle nociceptors in the head and neck regions, whereas muscle relaxation might not be an important part of the anti-migraine effect.

## 1. Introduction

OnabotulinumtoxinA (BoNT-A) is effective for the prevention of chronic migraine (CM) when injected in pericranial muscles [1,2,3]. As preventive treatment is markedly important for individual patients in the management of migraine [4], better patient compliance through an improved explanation of the mechanism of treatment may benefit patients. The mechanism of action of BoNT-A in treating migraines has been subject to preclinical and clinical studies. It has been demonstrated that BoNT-A exerts an effect on peripheral nerve terminals of unmyelinated C-fibers but not thinly myelinated Aδ-fibers [5,6]. BoNT-A cleaves the SNAP-25 protein after endocytosis, which inhibits both the release of signaling molecules, including calcitonin-gene-related peptide (CGRP), and the insertion of ion channels in the synaptic membrane, which is suspected to be part of the antinociceptive effect in the treatment of migraine [6,7]. Extracranial injections of BoNT-A may also inhibit intracranial meningeal nociceptors due to sensory fibers innervating both pericranial muscles and meningeal nociceptors via sutures of the skull [8,9].

BoNT-A is suggested to exert its inhibiting effect on nerve endings originating from neurons in the trigeminal and cervical ganglia and spread throughout injected muscles [6]. This reduces pain signals to the brain and prevents the activation and sensitization of central neurons associated with migraine chronification.

Patients with migraine have been reported to have increased pericranial muscle tenderness as well as more frequent neck pain compared to controls [10,11]. Moreover, patients with migraine and neck pain have been shown to have increased neck muscle stiffness interictally [12] and during migraine attacks. Neck discomfort and stiffness are also very common [13,14]. BoNT-A is known to block the presynaptic release of acetylcholine at the neuromuscular junction, resulting in muscle relaxation. Clinical trials have demonstrated that BoNT-A effectively reduces muscle contractions and pain in the treatment of cervical dystonia and spasticity, leading to its application in various other conditions characterized via abnormal muscle contractions [15,16,17,18]. There has been speculation about the role of the muscle relaxant effect in partially mediating the effects of BoNT-A in chronic migraine.

The study, therefore, aimed to explore whether the effect of BoNT-A in chronic migraine is achieved due to reduced pericranial muscle stiffness and nociceptive activity.

## 2. Methods

### 2.1. Study Population and Study Design

This was a six-month longitudinal prospective study observing two cycles of BoNT-A for the treatment of CM. The muscle stiffness, pressure pain thresholds, and pericranial tenderness scores were measured on the first day of the second cycle and at a mean of 5.6 weeks (ranging from 4.9 to 7.4 weeks, with 68% visiting in the 6th week) in the second cycle. Additionally, the daily intensity of headache and neck pain through both cycles were registered in headache calendars. We included BoNT-A treatment responders with CM, which at the Danish Headache Center is defined as at least a 30% reduction in the number of migraine days per month after BoNT-A treatment.

**Figure 1:**
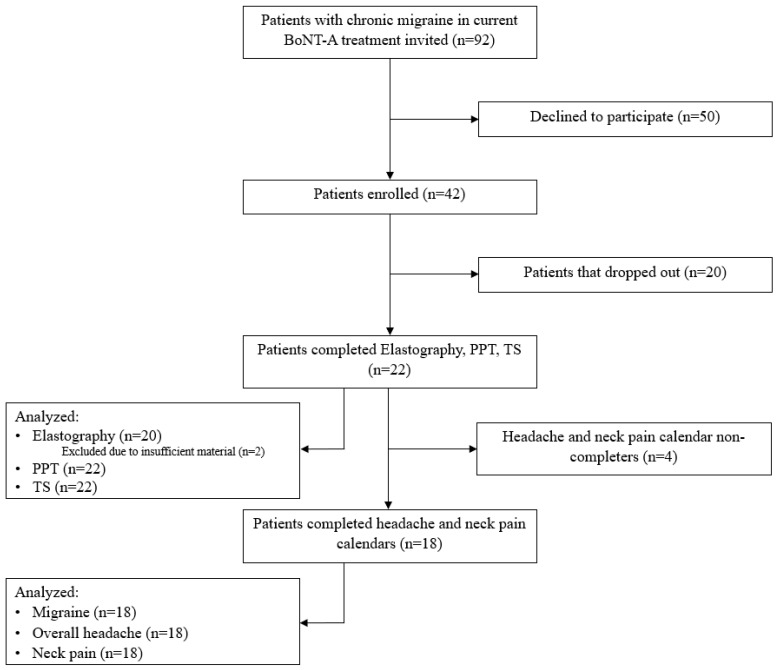
Flowchart of the inclusion process.

A total of 92 patients were invited to participate in the study, but 50 of them declined the invitation. A total of 20 patients were accepted but dropped out during the study period, and 22 patients completed the study (Figure 1). The demographics of the non-completers can be seen in Table 1; we were unable to obtain the migraine characteristics due to the general data protection regulation (GDPR). The causes of drop-out can be seen in Table 2. All participants provided written consent after receiving detailed oral and written information.

The study was conducted at the Danish Headache Center in accordance with the Declaration of Helsinki with later revisions. This study was approved by both the Regional Health Research Ethics Committee of the Capital Region of Denmark (H-20074198) and the Danish Data Protection Agency.

### 2.2. Inclusion and Exclusion Criteria

Patients were 18–65 years old and had at least completed three cycles, i.e., 9 months of BoNT-A treatment at the Danish Headache Center. The CM diagnosis was in accordance with the third edition of the International Classification of Headache Disorders (ICHD-3) [19]. Patients were excluded if they had medication overuse headaches, changed pharmacological preventive medication during the study period, or had any known cervical disorders, such as cervical spine disc herniation, whiplash, or spinal stenosis.

### 2.3. Treatment

Preventive treatment was performed according to the PREEMPT protocol [3]. Patients were injected at 31 locations in head and neck regions with 155 units of BoNT-A (Botox^®^, Abbvie/Allergan, Dublin, Ireland), with the option of further adding 5 units ± 8 using the follow-the-pain strategy [3] in cycles of 12 weeks.

### 2.4. Demographic and Clinical Characteristics

At the time of inclusion, patient information was collected considering age, sex, and level of physical activity on a scale from 1 to 4 (1 = regular high-intensity training; 2 = regular exercise sports at least four hours per week; 3 = walking or only light exercise; and 4 = sedentary lifestyle), height, weight, and years of migraine and the number of previous treatments with BoNT-A (Table 1).

### 2.5. Muscle Stiffness

Trapezius muscle stiffness was measured using ultrasound shear wave elastography. This works by measuring the speed (meter/second) of the shear waves propagating through a cross-section of the muscle. The Logic E9 scanner, Ge Healthcare (Chalfont St Giles, UK) with a linear 9 MHz ultrasound probe (9L) was used. Measurements were performed in the middle of the BoNT-A injection sites of the trapezius muscle (G1) according to the PREEMPT protocol. The measurements were conducted as previously described [12]. All data were saved as images by the observer and subsequently assessed by a trained observer blinded to the patient ID and sequence of the scans (JH).

### 2.6. Pressure Pain Thresholds

The pressure pain threshold (PPT) was assessed bilaterally on the trapezius and temporalis muscles and unilaterally on the dorsum of the non-dominant second finger corresponding to the middle phalanx (extracephalic region) [20,21]. The location of measurement on the trapezius muscle corresponded to the middle BoNT-A injection site (G1), and the location of measurement on the temporalis muscle corresponded to 3 cm superior to the middle point of a horizontal line between the lateral canthus of the eye and the ear. The PPT was defined as the minimal pressure where the sensation of pressure changed to pain. Three PPT measurements were performed at each location with a 30 s interval between two consecutive measurements, and the average of these was used as the final result. The electronic pressure algometer (Somedic Algometer type 2, Sollentuna, Sweden) was utilized to measure PPTs.

### 2.7. Pericranial Tenderness via Manual Palpation

Eight paired pericranial muscles and tendons were palpated with small rotating movements in a standardized manner and pressure bilaterally by a trained observer. The eight examined areas were the frontalis muscle, temporalis muscle, coronoid process, masseter muscle, mastoid process, sternocleidomastoid muscle, trapezius muscle, and neck muscle insertions. Subjects were asked to rate the tenderness from each location on a 4-point scale (0–3). Scores from each location were added to get a total tenderness score (TTS) ranging from 0 to 48. The method is reliable for testing pericranial tenderness [22]. The tenderness score was then divided into three groups: the cephalic tenderness score (CTS), the neck tenderness score (NTS), and the pain side score. Thus, these scores ranged from 0 to 24. The first is characterized by being sensorily innervated by the trigeminal nerves, and the second is innervated by nerves originating from C1 to C3. Measurements were grouped by pain side in the 15 patients with unilateral migraine, while an average of the two sides was used as a measurement for the migraine patients with bilateral headaches.

### 2.8. Headache and Neck Pain Assessment

Patients recorded the daily intensity of migraine, tension-type headache, and neck pain through two cycles of BoNT-A by filling out a daily headache and neck pain calendar on the maximum intensity of the day. Due to the individual planning of patients, the length of the individual cycles varied between 11 and 15 completed weeks. The intensity of the headache was measured on a 4-point scale from 0 to 3, which corresponded to no headache (0), mild headache (1), moderate headache (2), and severe headache (3), respectively [23]. Self-reported neck pain was assessed in a similar manner to current self-reporting guidelines for headaches [23]. Migraine, overall headache, and neck pain load were assessed as the clinically meaningful product of the frequency and daily intensity within a period of 7 days. Hence, the disease load ranged from 0 to 21. A score from 0 to 7 would correspond to sporadic days with a mild headache, and 7–14 would correspond to a daily headache varying between mild and moderate intensity or sporadic days with severe headache. A score from 14 to 21 would correspond to a daily moderate to severe headache. The product was, therefore, directly correlated with the monthly number of migraine days and headache, respectively, but a weekly score was preferred to more accurately show the daily differences. Overall, headache intensity was the sum of migraine and tension-type headaches, and in the case of a migraine day, a tension-type headache was defined as being absent.

### 2.9. Statistics

Results are presented as mean ± SD for muscle stiffness, PPTs, and TTS, and as a median with associated IQR for the load of headache and neck pain. The primary efficacy parameters were trapezius muscle stiffness, trapezius-, temporalis-, finger- PPTs, and TTS. The Shapiro–Wilk test and Q-Q plots were used to check that the difference between before and after muscle stiffness, PPTs, and TTS was normally distributed. All differences had at least a *p*-value of 0.15 when analyzed with the Shapiro–Wilk test and were, therefore, not close to raising suspicion of non-normality of data. Changes were compared using a paired T-test. The average of the two sides was used as a measurement when analyzing changes in PPT and muscle stiffness (elastography). Changes in headache and neck pain load were analyzed using the Wilcoxon signed rank test between the last week before BoNT-A treatment and the disease load in the sixth-week post-treatment of the second cycle. A post hoc analysis was also performed with measurements grouped according to the patient’s report of a predominant pain side (left or right). A unilateral migraine was defined as at least two-thirds of attacks with one-sided pain, as performed by Hvedstrup et al. [24]. An average of the two sides was obtained in patients with predominantly bilateral pain locations. The level of significance was set at 5%, and data were analyzed using IBM SPSS statistics, version 28.

## 3. Results

### 3.1. Study Sample

A total of 22 patients completed the baseline and follow-up measurements. The patients were, on average, 44 ± 10 years old, and 91% were female (Table 1). Out of 22 patients, 18 completed the headache calendars, covering a total duration of six months. The results of the elastography measurements were based on 20 patients, as the measurement quality was considered insufficient for two of the patients.

### 3.2. Muscle Stiffness

**Figure 2:**
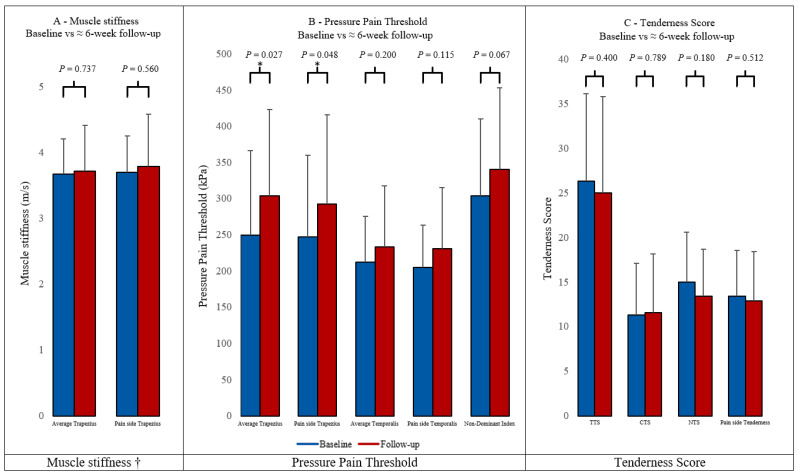
† The results of the elastography measurements are based on 20 patients, as the quality was insufficient for two of the measurements. The measurements were performed by a trained and blinded observer. The results correspond to the mean, and the error bars correspond to +1 SD. (TTS = Total Tenderness Score, CTS = Cephalic Tenderness Score, NTS = Neck Tenderness Score). *: *p* < 0.05.

There were no differences in trapezius muscle stiffness between baseline (3.68 m/s ± 0.53 m/s) and at 6 weeks in treatment (3.72 m/s ± 0.69 m/s) (*p* = 0.737). Similarly, no differences were found when comparing the muscle stiffness on the pain side at baseline and at the 6th week post-treatment (*p* = 0.560) (Figure 2A).

### 3.3. Pressure Pain Thresholds

The trapezius PPT increased from baseline (250 kPa ± 117 kPa) to 6 weeks after treatment (304 kPa ± 120 kPa) (*p* = 0.027). Similarly, when grouped by pain side, there was an increase in trapezius PPT from baseline (247 kPa ± 113 kPa) to 6 weeks (293 kPa ± 124 kPa) (*p* = 0.048).

There were no changes in temporalis PPT from baseline (212 kPa ± 63 kPa) to 6 weeks (233 kPa ± 84 kPa) (*p* = 0.200). Similarly, no changes were found when comparing the pain side at baseline and at the 6th week post-treatment (*p* = 0.115).

There were no changes in non-dominant index finger PPT from baseline (304 kPa ± 106 kPa) to 6 weeks (340 kPa ± 113 kPa) (*p* = 0.067) (Figure 2B).

### 3.4. Pericranial Tenderness by Manual Palpation

There were no changes in total tenderness score from baseline (26.3 ± 9.8) compared to 6 weeks in treatment (25.0 ± 10.8) (*p* = 0.400). Similarly, no changes were found when patients were grouped according to cephalic tenderness (*p* = 0.789), neck tenderness (*p* = 0.180), or the pain side (*p* = 0.512) (Figure 2C).

### 3.5. Headache and Neck Pain

#### 3.5.1. Migraine

**Figure 3:**
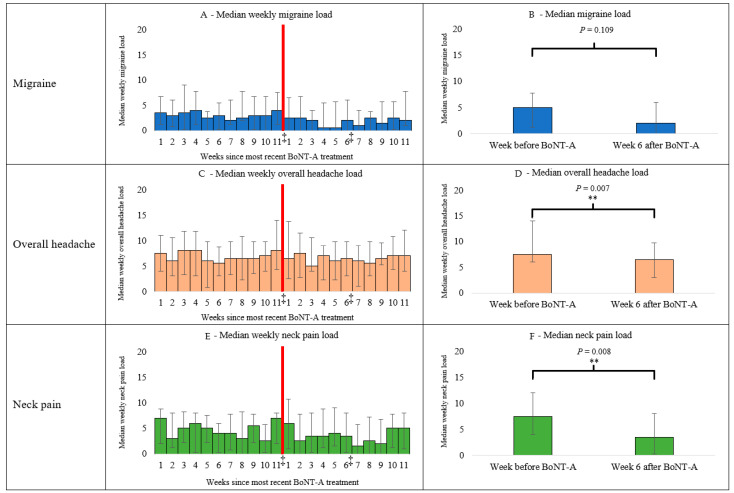
“‡” indicates the point of time of the muscle examinations in the BoNT-A cycles and the data used for (**B**,**D**,**F**). **: *p*< 0.01. The week before BoNT-A corresponds to the week before the redline, which is a different measurement than week 11 as patients had different cycle lengths due to individual planning. The results are based on the 18 patients who completed the headache diaries. The results of the headache diaries correspond to the weekly median value, and the error bars correspond to the IQR.

There were no changes in migraine disease load in the last week before BoNT-A (median: 5; IQR: 1.25 to 7.75) compared to after 6 weeks in treatment (median: 2.0; IQR: 0 to 6.0) (*p* = 0.109) (Figure 3B).

#### 3.5.2. Overall Headache

The overall headache disease load decreased between the last week before treatment (median: 7.5; IQR: 6 to 14) and the 6th week post-treatment (median: 6.5; IQR: 3.0 to 9.75) (*p* = 0.007) (Figure 3D).

#### 3.5.3. Neck Pain

BoNT-A decreased the neck pain disease load between the last week before treatment (median: 7.5; IQR: 4.0 to 12.0) and the 6th week post-treatment (median: 3.5; IQR: 0.25 to 8.0) (*p* = 0.008) (Figure 3F).

## 4. Discussion

The major findings of this study were that BoNT-A increased the trapezius PPT, but there were no changes in temporal PPT, finger PPT, pericranial tenderness, or trapezius muscle stiffness. Our study indicates that the mechanism of BoNT-A on CM is likely not mediated through the relaxation of the muscles as there were no changes in pericranial muscle stiffness observed. It is possible that the increase in trapezius PPT could partially be attributed to the desensitization of cutaneous and muscle nociceptors. These findings are in line with previous findings of a dissociation between pain and muscle relaxation in BoNT-A treatments in patients with cervical dystonia and spasticity [15,16,17]. The findings are also in line with the current understanding of the mechanism of action for BoNT-A in chronic migraine, as described in the introduction [6].

We found a numerical but not significant change in TTS after BoNT-A. This aligns with the study by de Tommaso et al. [25], who found a numerical but not significant change in TTS after a year of BoNT-A treatment for the 20 patients in their study. They also hypothesized that this served as evidence suggesting that the clinical efficacy of BoNT-A on CM was not mediated by pericranial muscle relaxation. Our results are further supported by the findings of a preclinical study by Gazerani et al. [26], which showed that BoNT-A injected in craniofacial muscles of rats reduced the local sensitivity of muscle nociceptors, possibly due to a decrease in the local interstitial glutamate concentration. Interestingly, Gfrerer et al. [27] found BoNT-A to alter the inflammatory gene expression and immune cells in the occipital periosteum but not in occipital muscle or fascia, which could suggest that the neck region is particularly sensitized in migraine. This agrees with our finding of a significant reduction in neck pain following BoNT-A treatment.

Another finding in this study was that BoNT-A seemed, on average, to stabilize the migraine disease load in patients, who on average had 11 previous treatments, as migraine disease load was generally low and stable around four throughout all 11 weeks, which equaled two days per week with a moderate migraine.

The present findings indicate that neck pain is attenuated by BoNT-A. The present study also suggests that there is no major wearing-off phenomenon in our patients with CM on stable BoNT-A treatment, which is in contrast with previous retrospective chart review studies [28,29]. The difference in results between this study and the study by Ruscheweyh et al. [28] could be explained by three major differences between the two studies. Firstly, the patients in our study had, on average, 11 previous treatments as opposed to a maximum of four. Secondly, we compared the last week before BoNT-A, which included patients who had only had 11 and 12 weeks since their last treatment as opposed to the minimum of 13 weeks since the last treatment. Thirdly, we measured the weekly disease load and not only frequency. The difference between the findings in our study and the study by Khan et al. [29] could be explained by the fact that we have grouped the results into a weekly based median value, which may mask single individual short-term exacerbations. However, in the current study, the results are similar to the results of Khan et al. when analyzing the effects of BoNT-A on overall headache and neck pain, as overall headache and neck pain disease load fluctuated more in relation to the BoNT-A cycles. The disease load in overall headache was around six, so the patients had a load comparable to two days with mild headache and two days with moderate headache each week.

### Limitations

A major limitation of our study is the fact that nearly 50% dropped out during the study period, which can have led to selection bias, though the two groups based on demographics were quite similar (Table 1). This, however, does not exclude the possibility of major differences in clinical characteristics between them. The sample size of 22 patients is also a limitation since it can lead to a lack of power in the study, which decreases the generalizability of the findings.

The cohort in this study was primarily women, and it has previously been shown that the response to BoNT-A show a small gender difference in favor of women [30]. Therefore, the present results are likely to be primarily relevant for only women with CM.

Another limitation is that the lack of change in muscle stiffness could be explained by the fact that the patients had had multiple treatments and were in a stable level of muscle stiffness, PPT, and tenderness. However, this seems unlikely since current evidence suggests that muscle relaxation of BoNT-A will reverse after 10–12 weeks. It would be highly interesting to repeat the study on BoNT-A-naïve patients. The present study uses both PPT and ultrasound shear wave elastography, which may not be available at other headache clinics. This may challenge the possibility of further studying the mechanisms behind the effects of BoNT-A in CM in other headache clinics around the world.

## 5. Conclusions

The increase in trapezius PPT, together with no changes in muscle stiffness or tenderness and decreased neck pain, suggest that the desensitization of cutaneous and muscle nociceptors specifically in the neck region, is part of the mechanism of action of BoNT-A on CM and that the muscle-relaxing effects might not be. This may lead to further research into the potential therapeutic applications of BoNT-A in other diseases.

## Figures and Tables

**Table 1:** Characteristics of the patients that completed the headache calendar and the patients that completed measurements of PPT, TS, and Elastography.

Demographic (SD)	Tests (Elastography, PPT, TS) (*n* = 22)	Headache Calendar (*n* = 18)	Non-Completers (*n* = 20)
Age (years)	44 (10)	43 (10)	43 (10)
Female sex	91%	89%	100%
Low level of physical activity *	64%	67%	55%
BMI	28 (5)	28 (6)	27 (6)
Unilateral Migraine	68%		
Percentage of days with neck pain		45%	
Migraine history			
Years since diagnosis	23 (12)	22 (11)	20 (10)
Number of BoNT-A treatments	12 (8)	11 (7)	13 (9)

* Low level of physical activity: Primarily sedentary activities or light exercise at least 4 h a week (category 3 and 4).

**Table 2:** Causes of non-completion (*n* = 20).

Causes	Number of Patients (% of Non-Completers)
Changed their mind	7 (35%)
Non-sufficient adherence to the study	7 (35%)
Changed migraine preventive treatment	2 (10%)
Debut of secondary disease	2 (10%)
Symptom exacerbation after PPT and TTS measurements	2 (10%)

## Data Availability

The data presented in this study are available on request from the corresponding author.
